# Effects of Nutrient Limitation on Antarctic Microbial Community Diversity, Composition, and Interactions: A Cryoconite Microcosm Study

**DOI:** 10.3390/microorganisms14081807

**Published:** 2026-08-16

**Authors:** Eli M. S. Gendron, Melia Scholl, Adam J. Solon, Rachel R. Rubin, Anna Bergstrom, Pacifica Sommers, Arvind Varsani, Steven K. Schmidt, Dorota L. Porazinska

**Affiliations:** 1 Department of Entomology and Nematology, University of Florida, Gainesville, FL 32611, USA; 2 Department of Ecology and Evolutionary Biology, University of Colorado, Boulder, CO 80309, USA; melia.scholl@colorado.edu (M.S.); a.solon@qmul.ac.uk (A.J.S.); rachel.rubin@colorado.edu (R.R.R.); pacifica.sommers@colorado.edu (P.S.); steve.schmidt@colorado.edu (S.K.S.); 3 Department of Geosciences, Boise State University, Boise, ID 83725, USA; annabergstrom@boisestate.edu; 4 The Biodesign Center for Fundamental and Applied Microbiomics, Center for Evolution and Medicine and School of Life Sciences, Arizona State University, Tempe, AZ 85287, USA; arvind.varsani@asu.edu; 5 Structural Biology Research Unit, Department of Integrative Biomedical Sciences, University of Cape Town, Cape Town 7925, South Africa

**Keywords:** algae, Antarctic Dry Valleys, cyanobacteria, diversity, networks, nutrient co-limitation, rotifera, trophic interactions, tardigrada

## Abstract

Dry, harsh, nutrient-limited environments such as the McMurdo Dry Valleys, Antarctica, may be particularly susceptible to changes in nutrient availability, demonstrating strong deterministic community assembly processes shaping communities within an ecosystem largely dominated by stochastic processes. Utilizing previously characterized cryoconite hole sediments from Canada Glacier in Taylor Valley, Antarctica, we carried out a microcosm study to examine the effects of alleviating nutrient limitation. Using a factorial experiment of nitrogen and phosphorus, we characterized the impact of nutrient additions on community growth, richness, composition, and interactions. We identified cascading effects where the addition of nutrients led to increased microbial phototroph growth (e.g., *Chlorococcum*) and abundance of higher trophic-level microbial and invertebrate predators (e.g., cercozoans and rotifers). Nutrient additions did not influence community richness but altered the composition and interactions within communities. Overall, nutrient additions promoted larger community networks dominated by positive inter-domain interactions, except for phosphorus additions, which resulted in communities dominated by negative interactions. Regardless of the type of interactions, all networks were dominated by cross-domain interactions. Unlike additions in other ecosystems, which resulted in changes to species richness, we show that nutrient additions in extreme oligotrophic environments can alter community composition and complexity, while maintaining richness.

## 1. Introduction

Microorganisms are key drivers of biogeochemical cycles across all ecosystems, and are responsible for approximately half of global primary production and all biological nitrogen fixation [1,2,3]. However, microbial functional traits are not completely interchangeable and microbial community composition can profoundly affect carbon and other nutrient cycling [4,5]. Understanding how microbial communities assemble is therefore important for predicting how ecosystems may respond to future global change projections [6,7]. Despite recent progress documenting microbial community composition across ecosystems, the underlying mechanisms that shape microbial community assembly remain poorly understood [8,9]. Among the important drivers of microbial community assembly is nutrient availability, especially in very cold and dry environments [10,11].

Nutrient availability is an example of a deterministic, bottom-up force that can directly and predictably constrain the rate and outcome of community assembly. However, stochastic processes, like priority effects or unpredictable disturbances, can override deterministic forces, especially in communities with small population sizes [12]. Communities in extreme environments such as glacial and peri-glacial environments may be especially susceptible to stochastic processes [9,10,13]. However, recent work examining microbial communities in cryoconite holes across the Taylor Valley of Antarctica has demonstrated clear patterns of spatial structuring, likely driven by valley-scale nutrient gradients. These communities become more diverse along a gradient of increasing nutrient concentrations [14,15], with the lowest diversity on the most inland and oligotrophic Taylor Glacier to the highest diversity on Commonwealth Glacier near the coast, and intermediate diversity on Canada Glacier, which lies between Taylor and Commonwealth glaciers [16,17].

Microbial activity on Antarctic glaciers is primarily found within cryoconite holes, shallow depressions that form from the solar-driven warming of supraglacial sediments melting into the glacier surface [18,19]. These holes contain liquid water and nutrients, creating isolated microhabitats capable of supporting microbial life [17,20,21]. The formation of ice lids in Antarctic cryoconite holes shields them from external inputs of organisms and nutrients [22]. Despite their small size, often less than a meter in diameter, cryoconite holes can cover 0.1–10% of glacial ablation zones [23,24,25], and thus significantly contribute to local carbon cycling [26] and nutrient fluxes [15]. Global change factors such as a warmer climate along with increased nutrient deposition could affect how these communities assemble and function.

Previous work characterizing the microbial communities of both natural and experimentally created cryoconite holes in Taylor Valley, Antarctica established that these communities are dominated by Cyanobacteriota and microalgae, yet they support diverse heterotrophic bacteria, Fungi, protists, and microinvertebrates [14]. Similar to microbial communities across the cryosphere, and throughout Antarctica in particular, these communities form the basis of nutrient cycling through a relatively simple food web [27,28,29,30,31,32] driven by shifting balances between microbial autotrophy and heterotrophy [33]. Because nitrogen (N) and phosphorus (P) availability in the Taylor Valley strongly co-vary with N and P concentrations in cryoconite holes, as well as with their productivity and microbial diversity [14,15], these communities are likely N- and/or P-limited and hence deterministically assembled. These findings prompt the question of how alleviating nutrient limitations could affect community diversity and activity within cryoconite holes.

Investigating the role of nutrient limitation in community assembly is important in the cryosphere because these habitats represent biologically active hotspots in an otherwise extreme and oligotrophic environment. The diversity patterns of natural cryoconite holes in Taylor Valley suggest that nutrient limitation likely prohibits many species from growing and being active components of communities. However, with a warmer climate and increased nutrient deposition, more species could grow, resulting in increased diversity and altered community dynamics. On the other hand, increased nutrient availability could favor opportunistic, fast-growing species and lead to reduced community diversity and complexity [34]. Moreover, it could disrupt established ecological interactions [35,36,37,38], increase competitive exclusion, and lead to the loss of sensitive taxa [39]. Understanding these dynamics in cryoconite holes can provide broader insights into how shifting nutrient availability can shape microbial ecosystems under extreme environmental constraints.

Thus, our overarching research question is the following: how does the availability of limiting nutrients, nitrogen (N) and phosphorous (P) affect microbial community assembly in Antarctic cryoconite holes? Our goal is to identify traits in community composition and interactions that can be indicative of changes in response to these variables. We conducted a nutrient addition microcosm experiment using cryoconite sediments from Taylor Valley. These sediments were incubated under four treatment conditions: N addition, P addition, combined N and P addition, and a water addition Control. We tested two main hypotheses. (H1) Alleviating nutrient limitation would enhance the growth of phototrophs, triggering a cascading effect by increasing the abundance of organisms at higher trophic levels. Alternatively, primary production and shifts in abundance of organisms across the food web would be limited by factors other than macronutrients. (H2) Nutrient enrichment would allow all present species to grow, thereby increasing detectable microbial richness, altering community compositions, and impacting potential species interactions. Alternatively, nutrient enrichment would allow opportunistic fast-growing species to bloom and outcompete slow-growing species, thereby decreasing community richness, and simplifying community complexity and interactions.

## 2. Materials and Methods

### 2.1. Microcosm Set-Up and Data Collection

For this nutrient addition experiment, we selected cryoconite hole sediments from Canada Glacier, Taylor Valley, Antarctica (Supplementary Figure S1). These cryoconite holes represent a middle ground along the Taylor Valley gradient of nutrient concentrations, organismal productivity, and diversity in the valley [17,27]. Thirty previously collected cryoconite sediment samples [15] were gently homogenized and then distributed among 20 sterile Petri dishes (60 mm diameter), each with 25 g dry weight equivalent and assigned to four experimental treatments. All microcosms were first equilibrated at 100% water holding capacity (WHC) by adding sterile water for one week before nutrient treatments were applied. Experimental treatments consisted of (1) +N sediments that received ammonium nitrate dissolved in sterile water to achieve 4 µmol N g sediment^−1^, (2) +P sediments that received sodium phosphate dibasic dissolved in sterile water to achieve 2 µmol P g sediment^−1^, (3) +NP sediments that received ammonium nitrate and sodium phosphate dibasic dissolved in sterile water to achieve 4 µmol N + 2µmol P g sediment^−1^, and (4) Control sediments that received the same volume of sterile water as nutrient treatments did, but no nutrients. Each treatment was replicated five times and incubated in a freeze–thaw growth chamber (Darwin Freeze/Thaw TH, Darwin Chambers, St. Louis, MO, USA) with temperatures cycling between 1 °C and 3 °C and light between ~100 Lux (5 h/day) and ~10,000 Lux (19 h per day) to imitate natural cryoconite hole conditions during austral summers. To keep the sediments from drying out and maintain their wet weight, the plates were watered on weekly basis. The experiment was terminated on the 84th day and subsamples from all experimental treatments along with five subsamples of the Initial sediment were sent to the University of Florida (Gainesville, FL, USA) for examination of microbial and invertebrate communities.

### 2.2. Phototrophic Growth and Metazoan Abundance

To evaluate shifts in phototroph growth (H1), we measured the proportion of each microcosm’s surface covered by all phototrophs and identifiable morphotypes (e.g., Cyanobacteriota in the genus *Nostoc*) on a weekly basis using the point intercept method [40]. To evaluate the effects of treatments on the higher trophic-level invertebrates (H1), they were extracted from ~20 g of sediments using modified Baermann funnels for ~48 h [41]. Extracted invertebrates were then counted under an inverted microscope (Zeiss Axio, Munich, Germany) and identified morphologically as rotifers and tardigrades. To preserve sediments from +N and +P treatments for another experiment, only Control and +NP treatments were evaluated for invertebrate count-based abundance.

### 2.3. Microbial Communities via Metabarcoding

To evaluate the effects of experimental treatments on cryoconite hole community richness, composition, and interactions, we extracted DNA from ~300 mg of sediments with a DNeasy PowerSoil Kit (Qiagen Inc., Germantown, MD, USA) according to the manufacturer’s protocols. Extracted DNA was amplified in triplicates using standard rRNA metabarcoding protocols from the Earth Microbiome Project (https://earthmicrobiome.ucsd.edu; accessed 12 August 2024). Briefly, Bacteria sequences were amplified using the 16S rRNA gene (515f and 926r) [42] and eukaryote sequences (Fungi, protists, metazoans) using the 18S rRNA gene (1391f/EukBr) primers [43]. Amplicons were visually confirmed using gel electrophoresis, triplicates pooled, and then they were sent to the Hubbard Center for Genome Studies, University of New Hampshire for multiplexing, TruSeq library preparation, and paired-end sequencing on an Illumina HiSeq 2500 (2 × 250 bp) (Illumina Inc., San Diego, CA, USA).

Raw reads were demultiplexed and then processed using R v4.3.2, primarily using the “dada2” v1.26 library and pipeline [44]. Primer regions were trimmed from the reads using “cutadapt” v5.2 Python scripts [45]. Paired-end reads were quality-filtered using the “filterAndTrim” function and sequence errors calculated using the “learnErrors” function. Reads were then dereplicated using the “derepFastq” function and sequence variants inferred, without pooling replicate samples, using the “dada” function. Lastly, reads were merged using the “mergePairs” function. The 16S rRNA (bacterial) sequences (represented by a total of 520,428 reads with an average merged length of 234 bp) and the 18S rRNA (eukaryotic) sequences (represented by a total of 707,966 reads with an average merged length of 134 bp) were used to produce community tables of amplicon sequence variants (ASVs). Both bacterial and eukaryotic ASV tables were filtered of sequences that were represented in negative Controls. To preserve the response of microbial communities to nutrient treatments via microbial growth and increase in abundance (Supplementary Figure S2A,B), ASV tables were not rarefied and instead all samples were standardized to the same amount of sediment (per 1 g) used for DNA extraction. This approach allowed us to preserve the full extent of taxonomic richness captured through sequencing (Supplementary Figure S3A,B). Taxonomy was assigned using the BLASTn v2.17 algorithm in conjunction with the ARB-SILVA v138.2 ribosomal sequence reference database [46,47]. We note that current discussions in taxonomy have resulted in changes to the names of key taxa referenced in the database used for analysis, specifically Proteobacteria/Pseudomonadota, Chloroflexi/Chloroflexota and Cyanobacteria/Cyanobacteriota. We opted to keep the original taxonomic strings to make it easier to reference back to the database we used, but wish to draw attention to the shift in nomenclature [48,49]. The bacterial community table was filtered for mitochondrial and chloroplast sequences and the eukaryotic community table for bacterial sequences. The eukaryotic table was further broken down into three separate tables of Microbial Eukaryotes (mostly protists), Fungi, and metazoans (mostly rotifers and tardigrades). Finally, rare ASVs (≤5 sequences across all samples) were removed from all community tables (1268 retained bacterial ASVs, 408 Microbial Eukaryote ASVs, 100 fungal ASVs, and 16 metazoan ASVs; Supplementary Table S2).

### 2.4. Nutrients and Biogeochemistry

To verify the original levels of nutrients and guide the establishment of nutrient treatments, we measured the total and organic C, N and P in the Initial sediment samples (Supplementary Table S1). We calculated the moisture content of each sample by weighing out 5 g of sediment, drying at 105 °C for 24 h and reweighing [50]. Total C, total organic C, and total N were determined by combustion on a Delta V Plus Isotope Ratio Mass Spectrometer (Thermo Scientific, Dreieich, Germany) with a 4010 ECS Elemental Analyzer (Costech, Milan, Italy), equipped with a Zero Blank autosampler (Costech, Milan, Italy) in the Boise State Stable Isotope Lab. Sediments were acid-fumigated prior to TOC analysis to remove inorganic C. Nitrate and ammonium concentrations were measured from 0.5 g of material extracted in 5 mL 2 M KCl. Samples were shaken for 30 min at 250 RPM, then filtered through a #42 Whatman filter paper before analysis for ammonium using the Seal Analytical method (EPA-148-C, equivalent to EPA method 350.1 version 2) on an AA500 continuous flow analyzer (Seal Analytical, Mequan, WI, USA), which uses an alkaline salicylate method with hypochlorite and sodium nitroprusside. For nitrate + nitrite, we used the Seal Analytical method EPA-132-C rev 1, which uses a sulfanilamide/NED cadmium reduction procedure, also on the AA500. Phosphate was measured from 1 g of material extracted in 3 mL 0.5 M NaHCO_3_ buffered to a pH of 10 with 10 M NaOH using the same procedure as the KCl extraction. After filtering, 10 mL of the extract was treated with 3 mL of 2 N HCl. Total organic P was measured as the difference in phosphate extracted in 1 N HCl before and after combustion of the sample. A total of 50 mL of 1 N HCl was added to 2 g of material and shaken for 16 h at 150 rpm. The samples were then centrifuged at 2000 rpm for 15 min using a Centra GP8 centrifuge (IEC, Needham Heights, MA, USA) and 10 mL of the supernatant was neutralized to a pH of 2 using 2 N NaOH. Phosphate from all extracts was measured using an acidic molybdate/antimony with the ascorbic acid reduction method (EPA-134-C, equivalent to EPA method 365.1) also on the AA500 instrument. Analyses were run in triplicate on each sample; however, we did not have enough sample mass to run total and organic phosphorous in triplicate. However, during the process of adapting the protocol to low mass weights, we found little variability.

### 2.5. Statistical Analyses

To address H1, the response of phototroph growth to all treatments (except Initial sediments) was tested using generalized linear models (GLMs) under default parameters, followed by pairwise comparison using estimated marginal means calculations to compare treatments using the base R function “glm” and the “emmeans” function from the “emmeans” v2.0.4 package [51]. The response of rotifer and tardigrade abundance to Controls vs. +NP treatments was tested using a *t*-test based on counts standardized to per 20 g of sediments.

To address H2, potential shifts in richness in response to treatments were analyzed using GLMs as above. Community compositional shifts were tested using the abundance-weighted Aitchison distance metric [52] and visualized using Principal Coordinates Analysis (PCoA) using the “pcoa” function in the “ape” v5.8 R library [53]. The overall effect of treatment on community composition was tested using PERMANOVA models under 999 permutations using the “vegan” v2.7 library function “adonis2” [54]. To compare individual treatments, post hoc testing was done using pairwise PERMANOVA models followed by Benjamini–Hochberg (BH) *p*-value adjustment for multiple testing. To further identify community shifts, the relative abundance of the 10 most abundant bacterial and eukaryotic taxa, of each treatment, were tested for significant effects of treatment compared to the Control. Due to the non-normality and interdependence of relative abundances, the effects of treatments were tested using the non-parametric Kruskal–Wallis and pairwise Wilcoxon tests followed by Benjamini–Hochberg (BH) *p*-value adjustment for multiple testing [55,56].

To infer shifts in potential microbial interactions, we performed co-occurrence network analyses for each treatment using the sparse inverse covariance estimation approach from the “SpiecEasi” v1.1.1 R package [57]. Prior to network analysis, community tables were filtered for taxa present in at least four of the five replicates. Since communities consisted of Bacteria, Microbial Eukaryotes, Fungi, and Metazoa, we utilized the cross-domain wrapper feature to control for amplification and sequencing differences across these different organismal groups [58]. The Meinshausen–Bühlmann’s neighborhood selection method with 50 subsamples was used to develop the network topology (taxa and interactions–nodes and edges) [59]. To better understand potential interactions among individual organismal groups, networks for each treatment were subsetted for the taxa of each organismal group (Bacteria, Microbial Eukaryotes, Fungi, and Metazoa). Networks were visualized and analyzed using the “igraph” v1.0.1 and “sna” v2.8 R libraries [60,61]. Network analyses focused on a subset of network metrics that focused on network complexity: network size (number of nodes and edges), degree (number of edges connected to a node), linkage density and connectance (edge strength weighted linkage density), cohesion (edge strength and relative abundance-weighted ratio of negative edges to positive edges), the ratio of edges to nodes, and modularity (the number of subcommunities and the extent of the overall community consisting of these subcommunities) [62,63,64]. We also evaluated the proportion of intra- and inter-domain interactions (i.e., Bacteria–Bacteria vs. Bacteria–Non-Bacteria interactions).

## 3. Results

### 3.1. Effects of Treatments on Phototroph Growth and Metazoan Abundance

**Figure 1:**
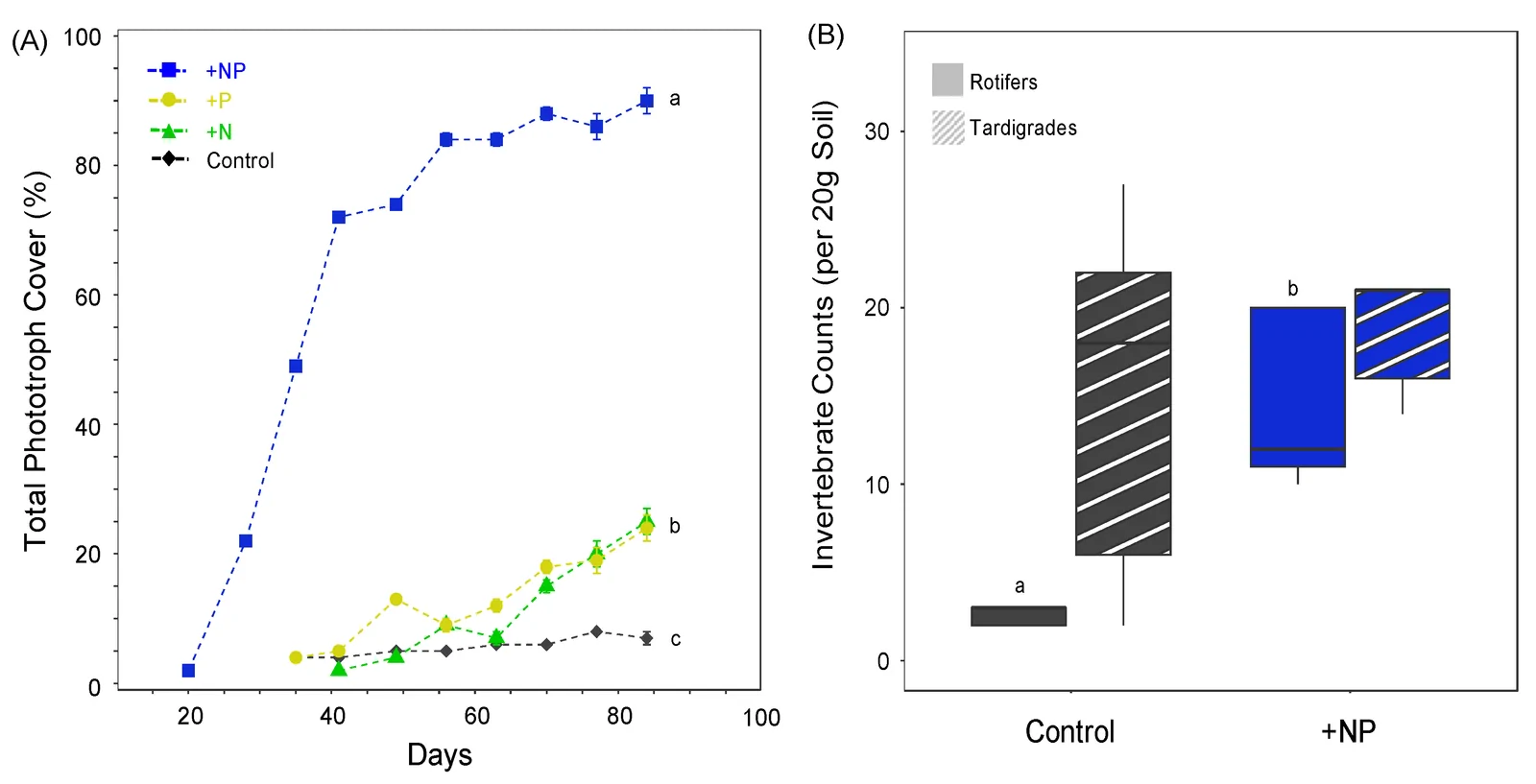
Responses of phototroph growth (**A**) and invertebrate abundance (**B**) to experimental treatments: 1. Control (water addition), 2. +N (nitrogen addition), 3. +P (phosphorus addition), and 4. +NP (combined N and P addition). Phototrophic growth points and error bars represent means and standard errors of five replicates. Relative abundance and invertebrate bars and whiskers represent means and standard errors of five replicates. The solid colors are rotifer counts and striped colors are tardigrade counts, with Controls as black and +NP as blue. Letters (a, b, c) indicate a significant difference between treatments.

In comparison to the Controls, all nutrient addition treatments significantly increased the growth of primary producers (Figure 1A; Supplementary Table S3). The +NP treatment resulted in the highest percentage of phototroph growth with an average final surface coverage of 90%. While both +N and +P treatments also led to greater growth than the Control, their final percent coverages were much lower (+N: 25%, +P: 24%). Notably, the +NP treatment was the first to exhibit any phototroph growth, beginning on day 20 of the experiment, whereas all other treatments did not show visible growth until around day 35. Further evidence of the combined effect of the +NP treatment on the growth of primary producers and cascading effects through the food web was observed in metazoan abundances, with significantly more rotifers found in the +NP treatments compared to the Controls (Figure 1B, Supplementary Table S3). The average tardigrade abundance did not significantly differ between +NP treatments and the Controls.

### 3.2. Effects of Treatments on Richness and Composition

**Figure 2:**
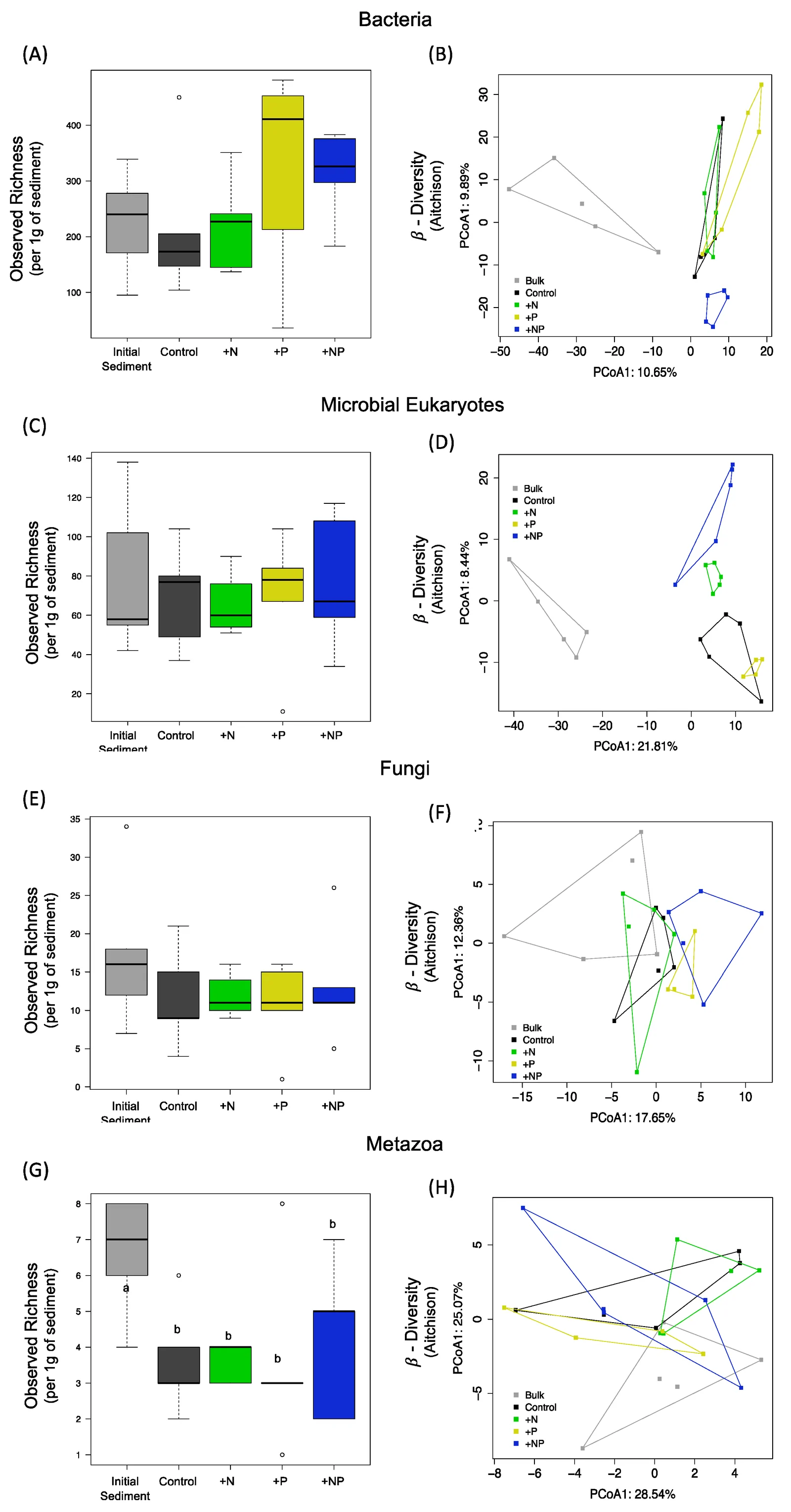
Response of richness and community composition to experimental treatments: 1. Control (water addition, black), 2. +N (nitrogen addition; green), 3. +P (phosphorus addition; yellow), and 4. +NP (combined N and P addition; blue), plus the Initial sediments (grey) for Bacteria (**A**,**B**), Microbial Eukaryotes (mostly protists; (**C**,**D**)), Fungi (**E**,**F**), and Metazoa (mostly rotifers and tardigrades; (**G**,**H**)). Bars and whiskers for richness represent means and standard errors of five replicates. Letters (a, b) indicate significant differences. Composition is visualized using PCoA plots based on Aitchison distance matrices. R^2^ indicates the proportion of community variation explained.

A comparison of ASV richness (as well as Shannon and Simpson’s indices) using GLMs revealed no significant effect of any treatments on richness (Figure 2) or diversity, and only a small effect of P on the evenness of the Microbial Eukaryote and fungal communities (Supplementary Table S4). The richness of microbial groups (Bacteria, Microbial Eukaryotes, and Fungi) remained similar to that of the Initial sediments (Figure 2A,C,E; Supplementary Table S4), whereas metazoan richness was lower in all experimental groups, including the Control, than in the Initial sediments (Figure 2G, Supplementary Table S4).

Despite the lack of an effect of nutrient addition on ASV richness, the community composition of each microbial group significantly changed with nutrient addition (PERMANOVA for Bacteria: R^2^ = 0.260, *p* = 0.001; Microbial Eukaryotes: R^2^ = 0.377, *p* = 0.001; Fungi: R^2^ = 0.273, *p* = 0.001) (Figure 2B,D,F, Supplementary Table S5). Pairwise comparisons specifically showed that bacterial community composition differed significantly between +NP and the other experimental conditions (Control, +N, and +P), which all differed from the communities in the Initial sediments. The pairwise comparisons of Microbial Eukaryote community composition showed that each experimental treatment was distinct from one another and from the Initial sediments, with the exception of +P and +NP communities, which were similar. The fungal communities were also distinct from one another, with the exception of Fungi in the +N treatment, which were sufficiently variable so that their composition also resembled both Control and +NP communities. Finally, the metazoan community composition showed no significant effects of treatments (*p* = 0.118) (Figure 2H, Supplementary Table S5).

**Figure 3:**
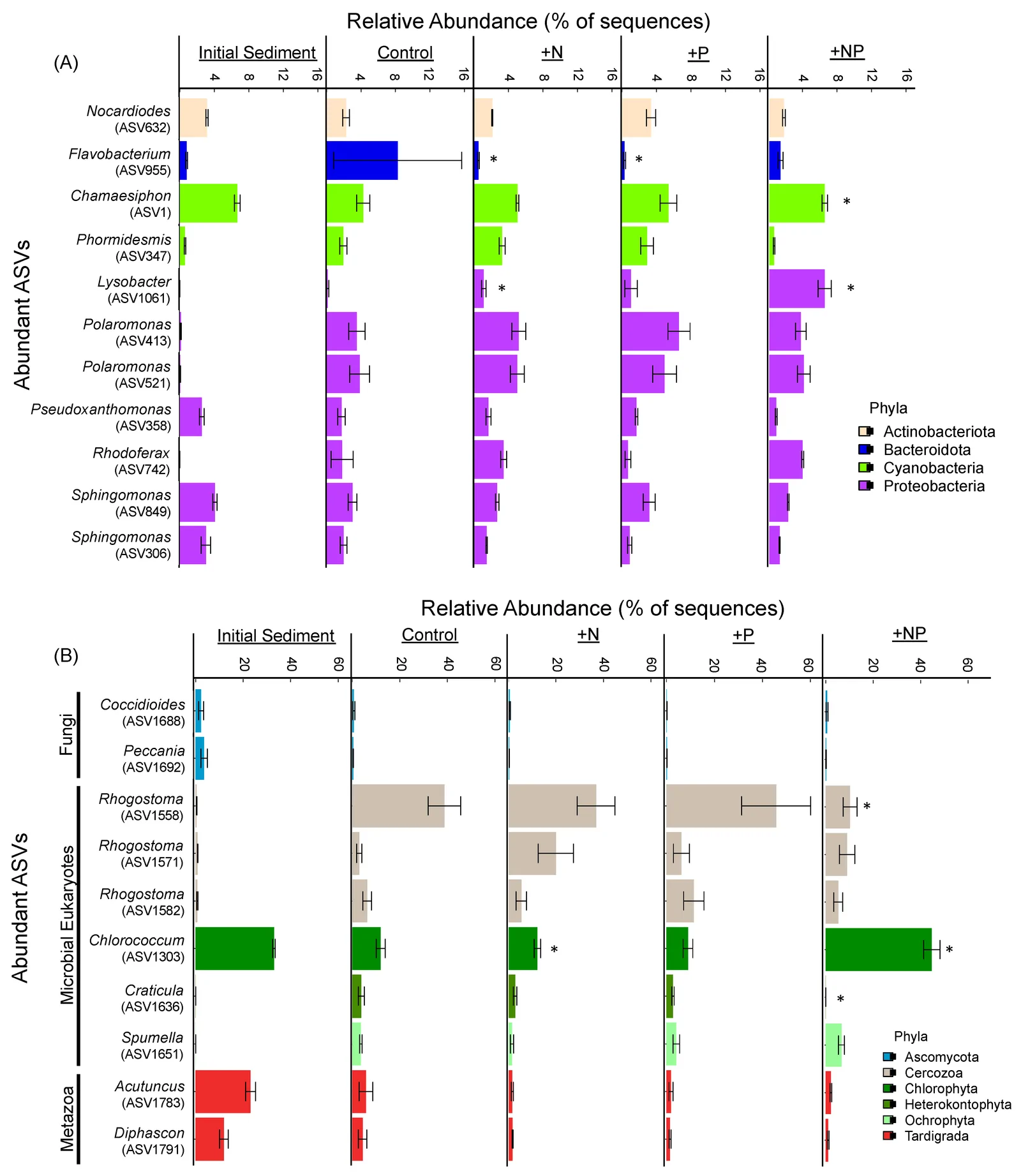
The relative abundance (% of sequences per treatment) of the most abundant bacterial (**A**) and eukaryotic taxa (ASVs; (**B**)) in Initial sediments (untreated) and experimental treatments: 1. Control (water addition), 2. +N (nitrogen addition), 3. +P (phosphorus addition), and 4. +NP (combined N and P addition). Bars represent means and whiskers represent standard errors of five replicates. Stars (*) indicate a significant difference from the Control samples.

Differences in the relative abundances of microorganisms provided a more specific view of some of the effects of nutrient treatments on community compositions. For example, the Initial sediments were unique in that Actinobacteria and Ascomycota were particularly abundant; the Controls were unique in their high relative abundance of Bacteroidota; and the +NP treatments stood out for their diversity of Proteobacteria/Pseudomonadota (Figure 3). The effects of nutrient additions were also particularly evident via higher relative abundances of Cyanobacteria/Cyanobacteriota from the *Chamaesiphon* genus (Figure 3A) and chlorophytes from the genus *Chlorococcum* (Figure 3B) in the +NP treatment compared to the Control. In addition, we observed higher relative abundances of cercozoan *Rhogostoma* in +N and +P treatments compared to Controls (Figure 3B, Supplementary Table S6). Finally, in comparison with the Initial sediments, tardigrade genera of *Acutuncus* and *Diphascon* were present at significantly lower relative abundances across all experimental treatments, including the Control (Figure 3B).

### 3.3. Effects of Treatments on Community Complexity

**Figure 4:**
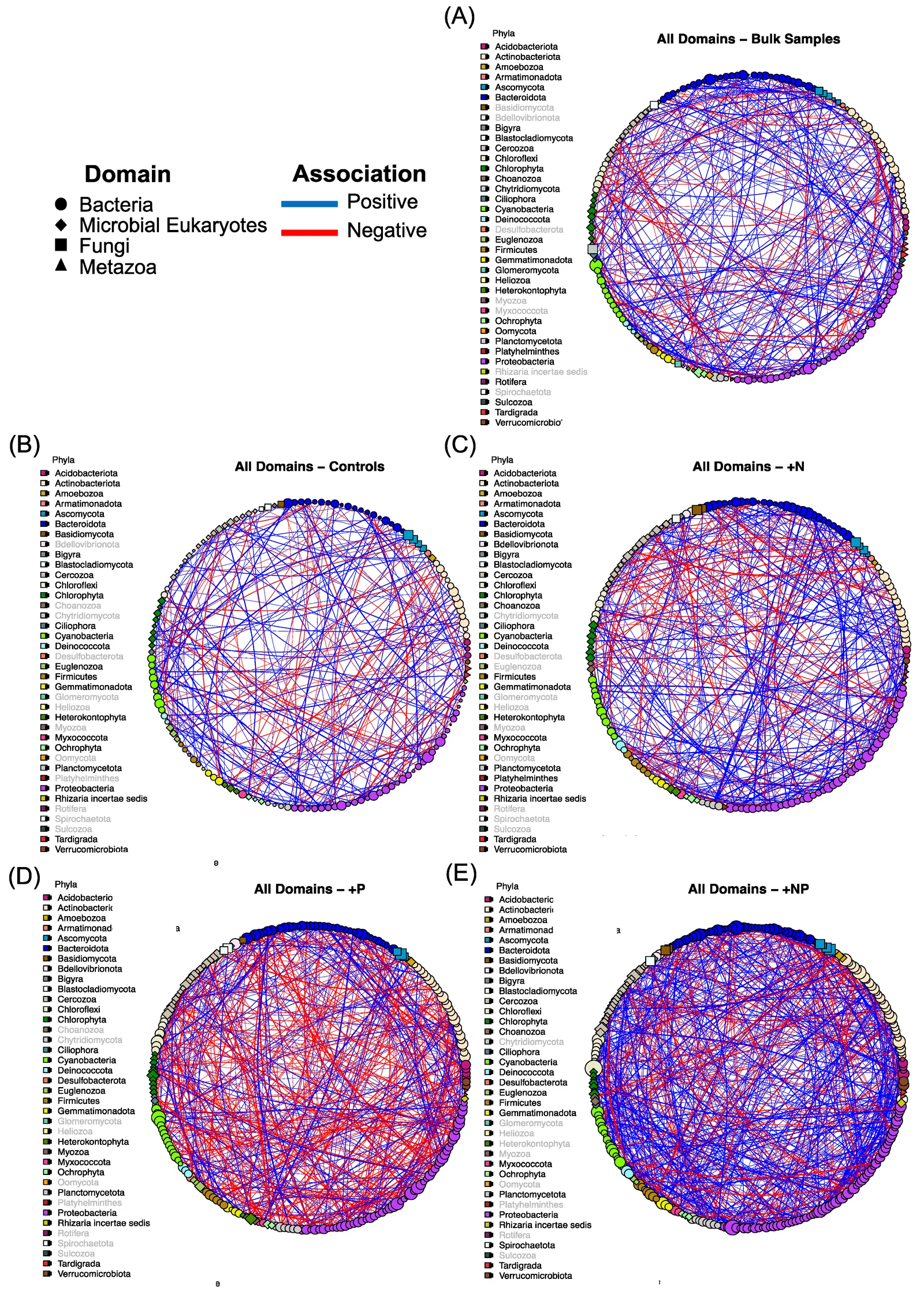
Cross-domain networks in Initial sediments (untreated; (**A**)) and experimental treatments: Control (water addition; (**B**)), +N (nitrogen addition; (**C**)), +P (phosphorus addition; (**D**)), and +NP (combined N and P addition; (**E**)). Larger nodes represent higher node degrees. Grayed-out phyla in the legend indicate an absence from the corresponding network. Green nodes represent phototrophic taxa, red nodes top metazoan consumers, and other colors denote specific microbial heterotrophic phyla.

The +NP treatment had the largest network (total number of nodes and number of edges) within the cross-domain community, containing the highest number of associated taxa (259 nodes), and the highest number of interactions among said taxa (718 edges) (Figure 4, Supplementary Table S7). The +P treatment network closely followed the +NP treatment in size, with 225 taxa and 587 interactions. The smallest network was the Control network, which contained the fewest taxa (147 nodes) and the lowest number of interactions (284 edges). Initial sediments and the +N treatment networks contained nearly identical numbers of taxa (176 and 175, respectively); however, the +N treatment network contained more interactions (Control: 359; +N: 410).

As networks got larger, they also demonstrated some evidence of becoming more complex. The ratio of edges to nodes increased with nutrient treatments relative to both the Control and Initial sediments (Supplementary Table S7). The Control and Initial sediments showed the lowest ratio (1.93 and 2.04, respectively), followed by single nutrient additions (2.34 and 2.61 for +N and +P, respectively), and then +NP treatment (2.77). The increase in the number of interactions was also reflected in the average node degree which ranged from an average of 3.86 edges/node in the Control to 5.22 in the +P treatment, and to 5.54 in the +NP treatment. However, while nutrient treatments resulted in more nodes with more edges on average, they did not result in an appreciable difference in network linkage density and connectance. Moreover, network size had no relationship with network modularity as all networks were highly modular (~0.71) and consisted of multiple subcommunities (Supplementary Table S7). The number of predicted subcommunities was 11 in the Control and +N treatment and 13 in the Initial sediment, +P, and +NP treatments.

In addition to shifts in network complexity, the nature of the interactions of community members was also affected by treatments. While most treatments identified cross-domain communities as primarily having mutually positive interactions between pairs of taxa (66–69% positive interactions), the +P treatment network predicted a community with a majority of negative interactions (56% negative interactions) (Figure 4; Supplementary Table S7). Furthermore, while positive cohesion (relative abundance and edge-weighted value of network connectivity across positive interactions) remained largely stable across treatments, total cohesion (positive + negative cohesion) and the cohesion ratio (ratio of negative cohesion: positive cohesion) were higher in the +P treatment network (total: 0.65 and ratio: 1.37, respectively) and the +NP treatment (total: 0.40 and ratio: 1.01 respectively). These values were largely driven by high negative cohesions focused within the +P (−0.38) and +NP treatments (−0.20).

Lastly, all networks were dominated by cross-domain interactions between taxa (inter-domain interactions) with within-domain interactions being much less common (intra-domain interactions; Supplementary Figures S4–S6), and in some cases completely absent (Supplementary Figure S7). The highest percentage of intra-domain interactions were identified in the Initial sediment (36%), +P (36%), and +NP (35%) treatments compared to the Control (31%) and +N treatment (31%). This pattern persisted across the domain-specific networks with the greatest difference observed in the microbial eukaryotic networks (20% in +P treatment vs. 12% in the +NP treatment) (Supplementary Table S7).

## 4. Discussion

We tested two hypotheses with a microcosm experiment that alleviated nutrient limitations within the previously described microbial communities of Antarctic cryoconite holes. Aligning with our H1, when both N and P were added together, the abundance of phototrophs and metazoans (rotifers) increased along with cascading effects on the relative abundances of other taxa across the food web. Despite the fact that, contrary to our H2, the taxonomic richness of the communities remained unchanged by nutrient addition, community compositions shifted to have more interactions and demonstrate more mutually positive relationships in those communities. Our findings highlight the multiplicative effect that alleviating co-limiting nutrients has on community assembly, composition, and potential relationships among taxa.

Our first hypothesis proposed that alleviating nitrogen (+N) and phosphorus (+P) limitations would enhance primary production and trigger cascading effects through the food web, increasing the abundance of higher trophic organisms. This hypothesis was generally supported by our findings. Nutrient additions, especially the combined N and P (+NP) treatment, led to increased phototroph growth, as evidenced by having the highest percent surface coverage. Furthermore, enhanced growth of primary producers in the +NP treatment was also associated with increased rotifer abundances, suggesting a bottom-up trophic cascade through the food web. Although we were unable to show the response of invertebrates to single nutrient additions, it is likely their abundances increased as well, albeit at proportionately lower levels reflective of the phototrophic growth rates.

In addition to shifts in the primary producers and high-trophic-level consumers (rotifers), we observed cascading effects on the relative abundance of other consumer taxa within the food web in response to treatments. For instance, the thecate amoeba *Rhogostoma* was largely absent from the Initial sediments, but dominated in the Control, +N, and +P treatments, though not in the +NP treatments. The dominance of *Rhogostoma* in these nutrient experimental treatments may have been due to lower photosynthetic growth from only alleviating a single limiting nutrient combined with potential grazing, as some *Rhogostoma* species have been shown to feed on algae [65,66], while the decreased relative abundance of *Rhogostoma* under +NP treatment could be due to competitive exclusion by larger predators such as rotifers, which increased in abundance under nutrient-rich conditions. This could also be indicative of the phototrophic growth outstripping heterotrophic growth when nutrient limitation is alleviated [67,68].

The rapid phototroph growth and shift in heterotroph abundances suggest a shift from microbial heterotroph-dominated communities to communities driven by microbial phototrophs and higher trophic-level consumers as nutrient co-limitation is alleviated. This is supported by the results that rotifers were especially responsive to +NP enrichment relative to the Controls. Rotifers may serve here as indicators of nutrient enrichment, as has been shown in warmer ecosystems [69,70]. While we were limited to describing the impact of individual nutrient additions on the highest trophic community members (rotifers and tardigrades), where we would expect to observe intermediate cascading effects, these results still point to the coupled nature of microbial phototrophic growth and higher trophic-level growth.

Natural cryoconite holes in Taylor Valley experience particularly low levels of available phosphorus and nitrogen [15]. Since the primary source of nitrogen inputs comes from cyanobacterial N-fixation [15], we expected phosphorus to be the primary limiting nutrient not only to primary producers, but across the entire food web. Instead, the significantly greater phototroph growth in the +NP as compared to +P alone treatment supports co-limitation by both N and P. The early and sustained dominance of phototrophs in the +NP treatment indicates that combined N and P availability also accelerates autotrophic microbial activity and biomass accumulation in contrast to the alleviation of a single nutrient limitation. These findings align with recent studies in Arctic and Tibetan ice-dominated ecosystems, which have also reported widespread microbial co-limitation by N and P [71], indicating that such co-deficiency may be common in the cryosphere.

Our second hypothesis predicted that nutrient enrichment would increase microbial richness and alter community composition. The first component of our hypothesis was not supported by our data, because ASV richness for Bacteria, Microbial Eukaryotes, and Fungi did not significantly change in response to any treatments. The only group for which richness changed across treatments was the Metazoa, decreasing across all nutrient treatments compared to the Initial sediments, possibly due to the lower carrying capacities of the microcosms, leading to increased competitive exclusion of some taxa or a lower diversity of physical habitat structures in the microcosms than the natural cryoconite holes [72]. These findings challenge the assumption that increased resource availability universally promotes biodiversity and instead supports the idea that the relatively low microbial richness in extreme environments may be constrained by other factors such as dispersal limitation [73].

Dispersal opportunities into communities were limited by the experimental design, as the microcosms themselves were isolated from immigration, reflecting the ice-lidded nature of Antarctic cryoconite holes [23,74]. However, one might have expected loss of richness through the competitive exclusion of taxa unable to keep up with “weedier” taxa such as the *Chlorococcum* dominating the nutrient-amended microcosms. The lack of significant extinctions suggests that taxa not favored by the conditions during the experiment may have remained dormant rather than going extinct, as the cryoconite environment already selects for species well-adapted to surviving months of dormancy.

Cryoconite holes are not the only nutrient amendment studies to have resulted in no increase in microbial richness following nutrient additions [75]. In fact, previous work showed that the combined addition of N and P decreased eukaryotic richness in supraglacial sediments from the Toklat Glacier in central Alaska [40]. This is consistent with the idea that alleviating nutrient limitations can favor the growth of a few fast-growing taxa, leading to species being outcompeted and thus reducing overall richness [10]. While our study did not observe an increase in richness, we also did not observe a decrease, suggesting that opportunistic taxa had a limited capacity to outcompete and displace taxa less responsive to enrichment. This finding suggests that cryoconite communities may experience “storage effects” [73,76], where unfavorable conditions can drive taxa to enter dormancy instead of being outcompeted. This strategy could provide cryoconite communities with a mechanism to maintain their diversity in the face of global change factors.

Despite the stable richness, PERMANOVA analyses revealed significant shifts in community composition across all microbial groups due to changes in the relative abundance of some taxa. Overall, the microbial communities responded more strongly than metazoans, suggesting greater sensitivity to bottom-up Controls on the scale measured by this experiment. The alleviation of the co-limiting nutrients particularly reduced the relative abundance of Bacteroidota (*Flavobacterium* sp.) and Cercozoa (*Rhogostoma* sp.), and increased the relative abundance of Proteobacteria/Pseudomonadota (*Lysobacter* sp.), Chlorophyta (*Chlorococcum* sp.), and Cyanobacteria/Cyanobacteriota (*Chamaesiphon* sp.). [14]. While the previous work identified *Pleurastrum* sp. as the dominate algae in sediments of this glacier, many *Pleurastrum* species have been moved to the genus *Chlorococcum*, which is found in Antarctica streams [30] and in soils above Canada Glacier [77]. In fact, the *Chlorococcum* 18S rRNA gene found in this study is a 100% match to *Chlorococcum* in a gully that feeds directly onto the surface of Canada Glacier cf. [77]. The response of these photosynthetic organisms to the nutrient additions highlights their potential to facilitate trophic cascades as they form the foundation of the food web [27,28,31,66]. The decoupling of richness and composition implies that shifts in the functional roles of taxa can occur without changes in alpha diversity, which holds potentially important implications for cryoconite hole functionality, such as in soils where changes in community composition affect biochemical cycling even when richness remains constant [6].

Similar to cryoconite holes, the microbial communities of perennially ice-capped Antarctic lakes are structured by co-limiting effects of N and P availability, resulting in a bottle effect on microbial community distributions [78], and altering community functionality [10]. The shifts in compositions in our study support the idea that nutrient availability can still act as an environmental filter in cryoconite holes, favoring the growth of taxa with specific traits or nutrient acquisition strategies [29,75,79]; however, it might not necessarily result in the removal of traits from the community due to dormancy.

We had hypothesized that nutrient addition would lead to shifts in microbial interactions, reflecting potential changes in community relationships and functions. Our network analyses partially support this hypothesis. Nutrient enrichment, particularly the +NP treatment, resulted in the largest network, with the highest number of potentially interacting taxa (nodes) and interactions (edges), followed by +P treatment. The increased network size may provide a community with more resistance to perturbations [80]. However, while the +P treatment also produced a large network, the nature of the community interactivity differed markedly from the +NP treatment. Where the +NP network was dominated by mutually positive interactions (e.g., mutualism or facilitation), the +P network was dominated by negative interactions, suggesting increased competition or antagonism.

Phosphorus enrichment may have intensified competition by releasing previously P-limited taxa that now had to compete for other limiting resources (e.g., N, space, and light) [39]. In contrast, while the +N treatment did increase network size relative to the Control treatment, it did not differ substantially from the Initial sediment network and was much smaller than the +P treatment, providing further evidence that N is not the primary limiting nutrient. This aligns with previous findings that P is the ultimate limiting nutrient for microbial productivity in cold and dry environments [15,81].

This difference in interactivity between treatments underscores the role of nutrient stoichiometry in shaping microbial community dynamics [36]. High proportions of positive interactions, as observed in the Controls, +N, and +NP treatments, may be indicative of community stability, either through direct facilitation and cooperation [82] or through the mutual relief of limiting resources [83]. On the other hand, positive interactions could lead to instability by facilitating strong feedback loops that result in the mutual extinction of connected species, especially during heavy disturbances [84]. In contrast, higher rates of negative interactions have been found to be indicative of network stability [64,85].

The altered nature of microbial community interactivity, positive versus negative interactions, may also have contrasting implications for ecosystem dynamics. We observed an increase in both the number and proportion of negative interactions in the +P and +NP treatments, indicating increased competition under the alleviation of nutrient limitation. Sudden nutrient availability, especially the elimination of P limitation, in nutrient-limited environments can drive excessive or luxury uptake of P for intracellular nutrient storage [86]. Throughout the cryosphere, P luxury uptake is a recurring strategy across trophic levels, ranging from primary producers such as Cyanobacteriota and microalgae to higher trophic consumers such as microinvertebrates (e.g., tardigrades and rotifers) [87,88,89]. Luxury P uptake may be the mechanism by which the alleviation of nutrient limitation in cryoconite holes can lead to increased competition under resource availability, consistent with the stress-gradient hypothesis [90].

Lastly, while we observed a general trend in increased richness in the +NP treatments (at least for Bacteria), our results showed no significant differences across treatments. To explain the changes we observed in community composition, we can point to the modular nature of other aquatic Antarctic communities where communities of microbial taxa are organized into groups of functional significance [91]. We found all communities, including the Initial sediments, display similarly high modularity values, with similar numbers of predicted groups. The differential selection of subcommunities based on environmental factors such as nutrient availability can help explain the compositional shifts we observed without the loss of species from the overall species pool, especially when overall richness is relatively low [92]. Instead of directly competing with all members of the network, taxa are potentially able to co-exist through the niche-driven assembly of subcommunities [93].

## 5. Conclusions

In conclusion, our results demonstrate that nutrient enrichment affects not only phototroph growth, abundance, and composition, but also the structure and dynamics of their ecological interactions. The combined alleviation of N and P limitation fostered the largest and most cooperative networks of presumably interacting taxa, while adding P alone led to communities primarily structured by competitive interactions. These findings highlight the nuanced and context-dependent nature of microbial responses to nutrient inputs and suggest that, under current global change projections (e.g., increased nutrient availability and climate warming), microbial communities in the cryosphere will likely experience substantial shifts in their activities, diversities, and functions, with possible magnifying feedbacks to other environments (i.e., streams, lakes, and soils) that surround the glaciers they live on.

## Data Availability

The data presented in this study are openly available through the following sources. Raw sequencing read data is available from the NCBI under the following accession numbers: SAMN49550769-SAMN49550818. In addition, 16S and 18S metabarcoding ASV Table are also available from the USAP-DC repository under dataset ID 601957 and the DOI 10.15784/601957. Code used for analysis and the production of figures is available https://github.com/ems-gendron/Cryconite-Microcosm-Community-and-Network-Analyses; last maintained on 8 August 2026.

## Supplementary Materials

The following supporting information can be downloaded at: https://www.mdpi.com/article/10.3390/microorganisms14081807/s1, Figure S1: Map of Sample Sites; Figure S2: Sequence Abundance; Figure S3: Sequence Rarefaction Curves; Figure S4: Bacterial Subnetwork; Figure S5: Microbial Eukaryote Subnetwork; Figure S6: Fungal Subnetwork; Figure S7: Metazoan Subnetwork; Table S1: Biogeochemistry; Table S2: ASV Table; Table S3: Phototroph Abundances; Table S4: Alpha Diversity; Table S5: Community Composition; Table S6: Abundant Taxa; Table S7: Network Characteristics.
