# Supplementary material for: Effects of Nutrient Limitation on Antarctic Microbial Community Diversity, Composition, and Interactions: A Cryoconite Microcosm Study
Source: Microorganisms. 2026 Aug 16;14(8):1807. doi: 10.3390/microorganisms14081807 (PMC13515893; doi:10.3390/microorganisms14081807)
Supplement: Supplementary file 1 [file microorganisms-14-01807-s001.zip › Supplementary_Figures.pdf]

Figure S1

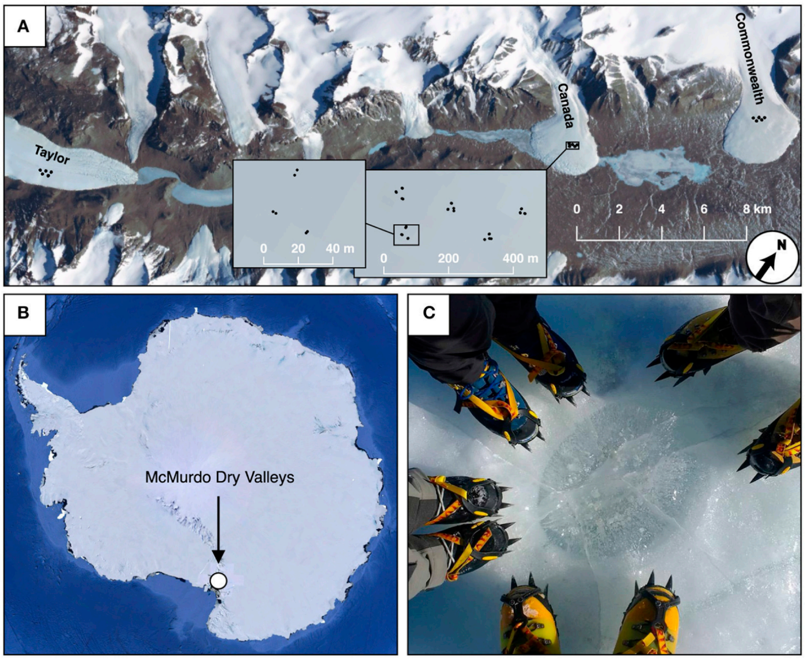

Supplementary Figure S1. Map of Sample Sites. Aerial view of McMurdo Dry Valleys, Antarctica (A) with glaciers and sample sites labeled, particularly for Canada glacier from which all samples came from, the location of the McMurdo Dry Valleys on the continent of Antarctica (B), and a close up of an iced over cryoconite hole on Canada glacier (C). This figure was taken wholly and unmodified from Darcy et al. 2018 who's work provided the sample material for this study.

Figure S2

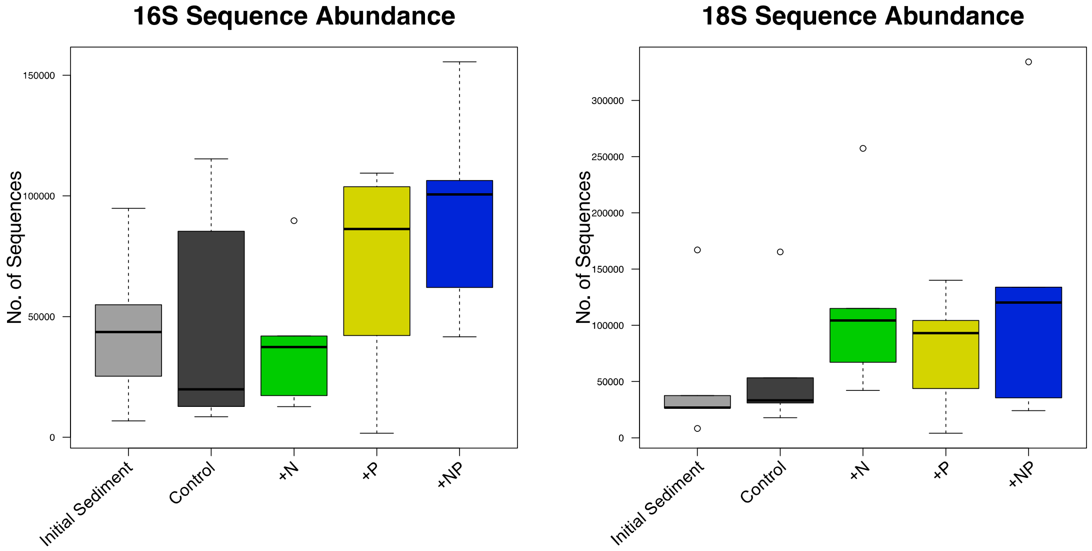

Supplementary Figure S2. Amplicon Sequence Abundance. Sequence abundance of 16S rRNA (A) and 18S rRNA (B) reads in Initial sediments and experimental treatments: 1. Control (water addition), 2. +N (nitrogen addition), 3. +P (phosphorus addition), and 4. +NP (combined N and P addition).

Figure S3

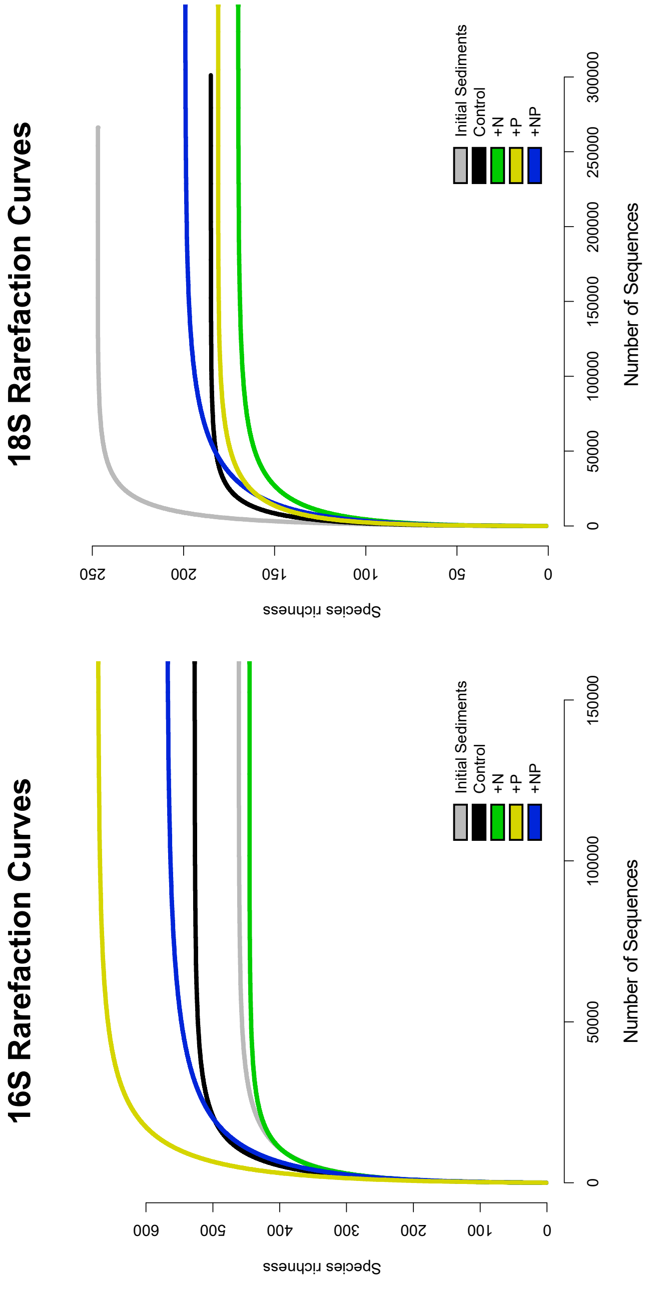

Supplementary Figure S3. Amplicon Sequence Rarefaction Curves. Rarefaction curves for 16S rRNA (A) and 18S rRNA (B) sequence data across Initial sediments and experimental treatments: 1. Control (water addition), 2. +N (nitrogen addition), 3. +P (phosphorus addition), and 4. +NP (combined N and P addition).

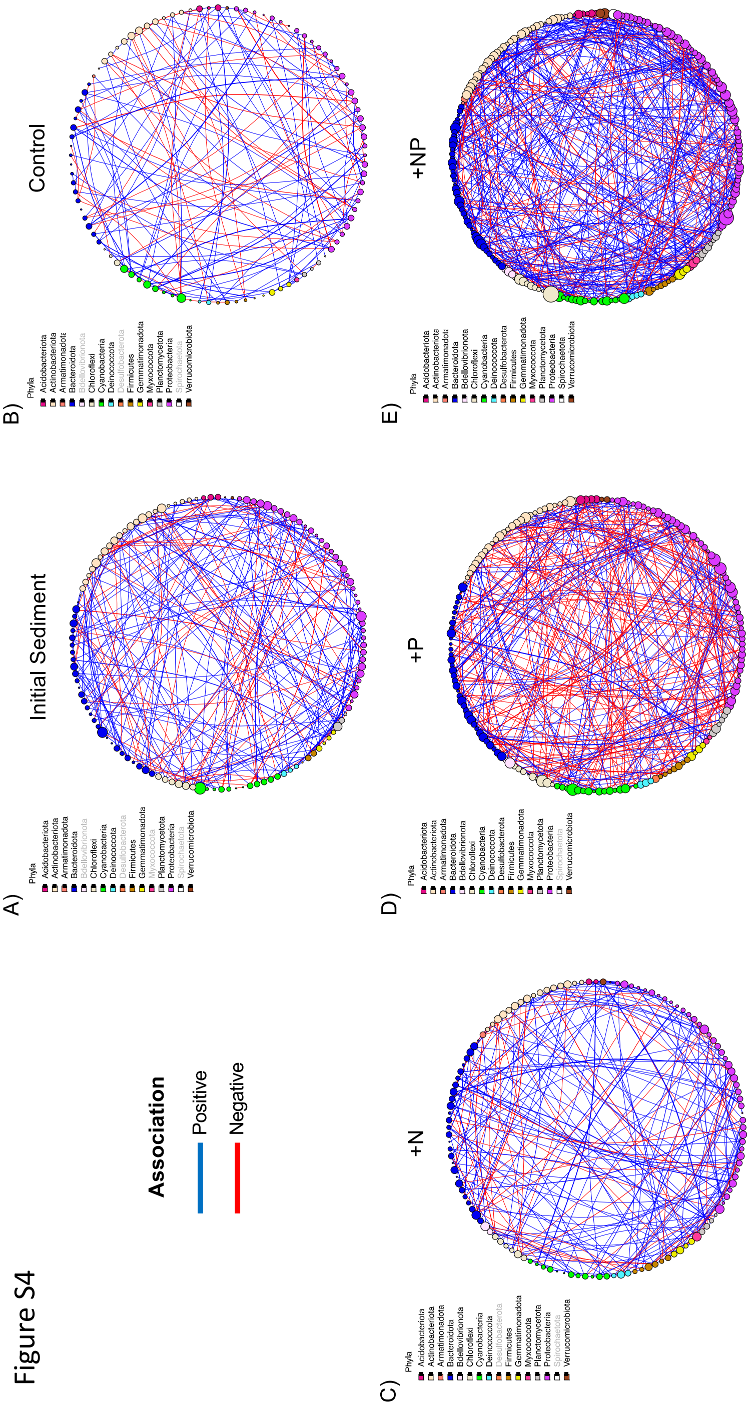

Supplementary Figure S4. Bacterial Subnetwork. Initial sediments (untreated; A) and experimental treatments: 1. Control (water addition; B), 2. +N (nitrogen addition) (C), 3. +P (phosphorus addition; D), and 4. +NP (combined N and P addition; E). Larger nodes represent higher node degrees. Greyed-out phyla in the legend indicate absence from the corresponding network. Green nodes represent phototrophic taxa and other colors denote specific heterotroph groups.

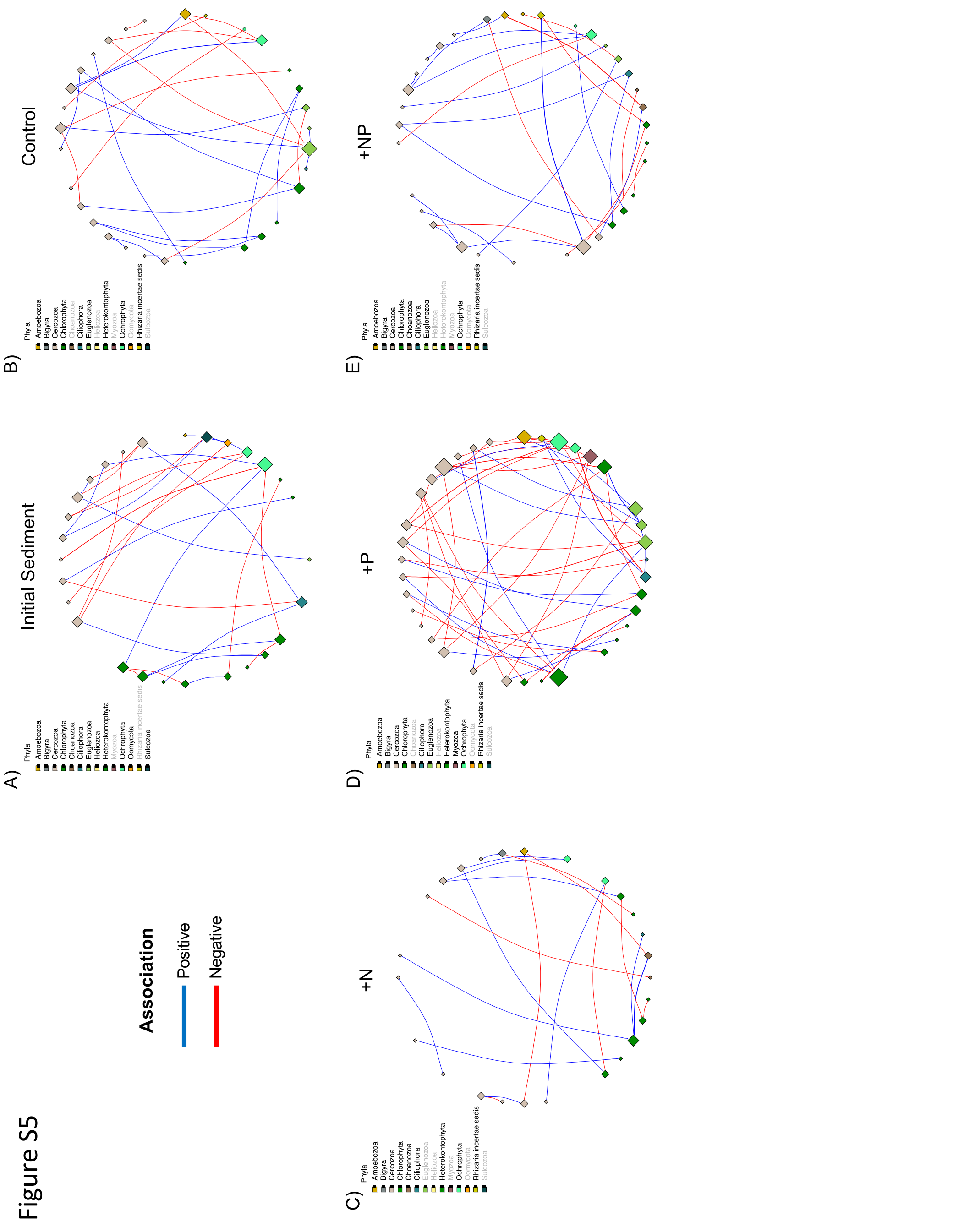

Supplementary Figure S5. Microbial Eukaryote Subnetwork. Initial sediments (untreated; A) and experimental treatments: 1. Control (water addition; B), 2. +N (nitrogen addition) (C), 3. +P (phosphorus addition; D), and 4. +NP (combined N and P addition; E). Larger nodes represent higher node degrees. Greyed-out phyla in the legend indicate absence from the corresponding network. Green nodes represent phototrophic taxa and other colors denote specific heterotrophs.

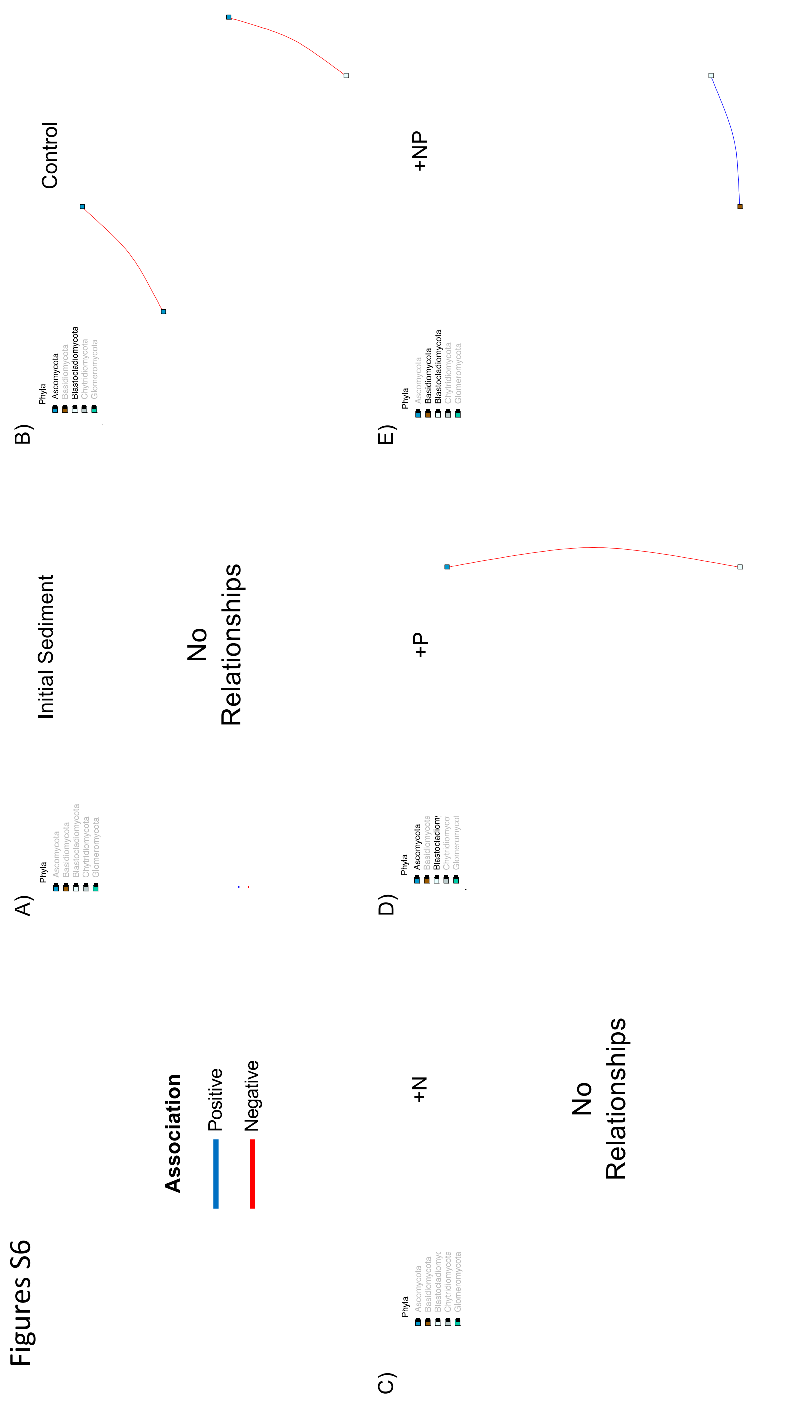

Supplementary Figure S6. Fungal Subnetwork. Initial sediments (untreated; A) and experimental treatments: 1. Control (water addition; B), 2. +N (nitrogen addition) (C), 3. +P (phosphorus addition; D), and 4. +NP (combined N and P addition; E). Greyed-out phyla in the legend indicate absence from the corresponding network.

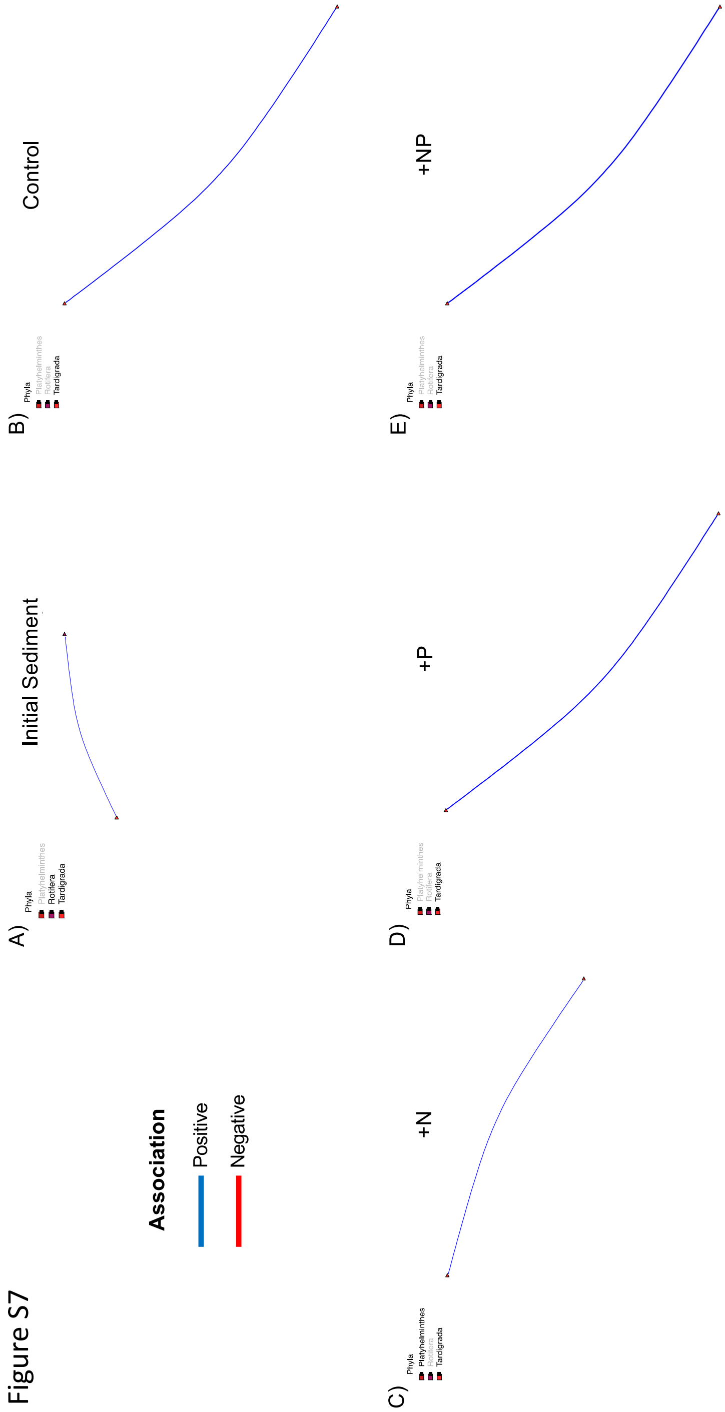

Supplementary Figure S7. Metazoan Subnetwork. Initial sediments (untreated; A) and experimental treatments: 1. Control (water addition; B), 2. +N (nitrogen addition) (C), 3. +P (phosphorus addition; D), and 4. +NP (combined N and P addition; E). Greyed-out phyla in the legend indicate absence from the corresponding network.
